# Healthy City Community Space-Oriented Structural Planning and Management Optimization under COVID-19

**DOI:** 10.3390/ijerph20053863

**Published:** 2023-02-21

**Authors:** Ya Yang, Zhengyu Jiang, Yawei Hou, Huaxing Wang, Zeyu Wang

**Affiliations:** 1 School of Architecture & Urban Planning, Anhui Jianzhu University, Hefei 230009, China; 2 School of Economics and Finance, Xi’an Jiaotong University, Xi’an 710061, China; 3 School of Mechanics and Civil Engineering, China University of Mining and Technology, Xuzhou 221116, China; 4 Law School, Hangzhou City University, Hangzhou 310000, China; 5 School of Economics, Zhejiang University, Hangzhou 310000, China; 6 School of Public Administration, Guangzhou University, Guangzhou 510006, China

**Keywords:** healthy city, health security, Community spatial structure planning, community space

## Abstract

This work studies ways of Healthy City Construction (HCC) and Urban Governance Optimization (UGO) during the COVID-19 pandemic. The specific urban community space planning structure is proposed following a literature review on the healthy city’s theoretical basis and historical development. Then, the proposed HCC-oriented community space structure is tested by surveying residents’ physical and mental health and infectious risk using a questionnaire survey and Particle Swarm Optimization (PSO). Specifically, the particle fitness is calculated according to the original data conditions, and the community space with the highest fitness is determined. Based on the calculation, the community space’s neighbors are investigated from different aspects through a questionnaire survey on patients’ daily activities and community health security coverage. The results showed that: (1) The score of daily activities of community patients with respiratory diseases was 2312 before the implementation of the proposed community structure and 2715 after the implementation. Therefore, the service quality of residents increases after implementation. (2) The proposed HCC-oriented community space structure improves the physical self-control ability of chronic patients and helps them reduce their pain. This work aims to create a people-oriented healthy city community space, improve the city’s “immune system,” and regenerate the energy and environmental sustainability of the urban living environment.

## 1. Introduction

Since early 2020, countless human, financial, and material resources have been invested in disease control and prevention with the outbreak of the Corona Virus Disease 2019 (COVID-19) pandemic. The pandemic has aroused panic among the population and has also rung an alarming bell against humans to protect their health better against diseases while pursuing economic development [1]. This is especially true for urban residents who live in extremely crowded community spaces with relatively fewer health facilities and inadequate medical services. Thus, the voice for a Healthy City Construction (HCC) is a rising concern for public health [2]. The HCC involves community space planning and intelligent management on a legal basis, among others. Its ultimate goal is to safeguard the resident’s health and safety and improve their living space and subjective well-being [3].

Urban management has always tried to improve residents’ health and has achieved some success and academic achievements. Over the years, scholars have found that the sustainable development philosophy is the key to HCC. Urban transport planning, public health facilities, and supportive policies are all essential for the HCC [4]. Yang et al. evaluated several Chinese cities using the Healthy City Construction Index (HCCI). The team explained that different urban districts and streets’ HCCI could be positively correlated, negatively correlated, or not correlated [5]. In China, HCC is just taking its maiden flight. Other studies examined residents’ mental states during the COVID-19 pandemic. They observed that the national lockdown policies had harmed the residents’ health, causing social distancing, loneliness, and eating disorders [6]. Another research group found the HCC played a major role in relieving residents’ mental stress during the COVID-19 pandemic. The research highlighted the potential and key aspects of urban space regeneration. The research results enabled people to live new social lives, helping people to heal disease with a better mindset and get through hard times [7]. There is also evidence that social relationships, physical vitality, life attitudes, diet, and weight are important health indexes. Creating an environment that supports healthy behaviors helps engage citizens and builds their confidence [8]. This further demonstrates the importance of HCC. In urban community spaces, structure planning and the residents’ physical and mental health are correlated [9]. In order to encourage residents’ physical exercise, the community spatial structure must consider both the individual household’s life habits and overall demographic distribution as much as possible. On the other hand, environment protection and public resource conservation can strengthen social cohesion and promote a green and open community space.

Therefore, this study will study the specific requirements of HCC-oriented community space planning and management and proposes an urban community space planning structure. The paper then discusses the residents’ physical and mental health under the proposed structure. Based on this, it summarizes the HCC development requirements and standards. In order to do so, based on the smart city residents’ health assessment index, a questionnaire survey is designed. Ultimately, the intelligent management measures of the smart and healthy city are explored, highlighting the importance of healthy city-oriented Urban Governance Optimization (UGO).

## 2. Methods

### 2.1. Theoretical Sources of Healthy and Smart Cities

The smart city is an information-based digital city development model by emerging network technologies such as the Internet, Internet of Things (IoT), and edge computing. Its main features are the integration of smart technologies with urban facilities and urban governance to generate a low-carbon and human-friendly living environment [10]. Based on the smart city model, the “healthy city” is a complex system of engineering. Thus, HCC intersects with the field of “smart city” construction [11]. An HCC-oriented community space planning focuses on people orientation. The healthy city is a new and future sustainable urban development paradigm in the Internet era. In urban governance, people orientation must be reflected in all stages, including urban planning, development, and construction, to achieve sustainable growth. A healthy city is expected to be a panacea for improving public social services and urban residents’ health and security [12]. Table 1 shows the difficulties of constructing a healthy city.

**Figure 1:**
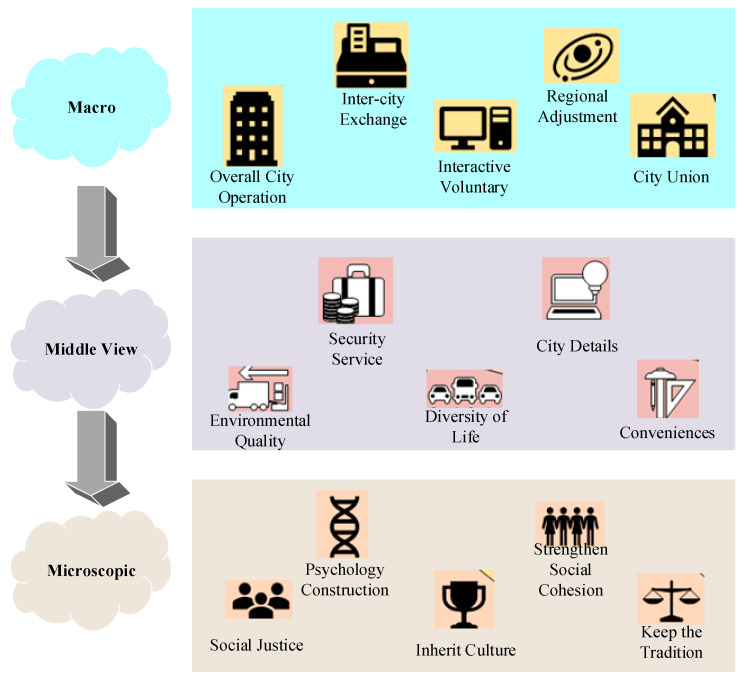
Three important structures of healthy cities.

HCC should consider the macro, meso, and micro factors. Then, in terms of residents’ health, HCC will involve socialized health, urbanized health, and population-based health. The specific structure is displayed in Figure 1.

According to Figure 1, theoretically, the HCC must meet the following requirements. The community space is the basic unit of a city and the main place for residents’ daily activities. Accessing health services and facilities is a community residents’ common demand [13]. A healthy city integrates, connects, and supplies various health service resources in the community and strengthens residents’ health-management awareness and ability. It also improves the community health services’ efficiency and effect [14,15]. HCC-oriented community spatial structure planning, management, and optimization provide a basis for evaluating the current situation of community health service security in China. They will help analyze the factors affecting the efficiency of community health service centers to formulate appropriate and effective measures. This has practical significance for solving the residents’ medical treatment difficulties and realizing China’s people-oriented medical reform goal.

### 2.2. Using Particle Swarm Optimization (PSO) to Evaluate Residents’ Health Status and Infectious Risk in a Healthy City

**Figure 2:**
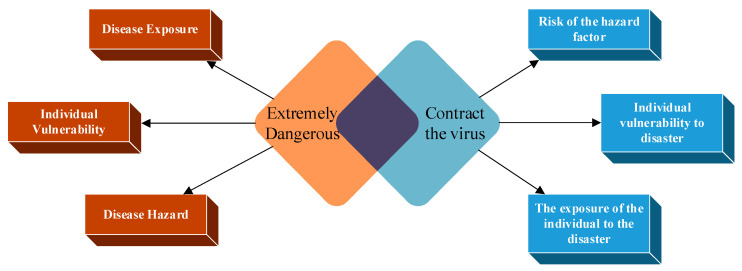
Residents’ primary infectious factors.

COVID-19 is a highly contagious respiratory infectious disease. The virus can spread directly or indirectly through the host. Three primary factors increase the resident’s infectious risk, as explained in Figure 2:

The disease’s infectious risk will be analyzed and calculated as follows: Define the risk value = R(A,T,V). *R* is calculated by Equation (1).

(1) R=H×E×V

In Equation (1), *R* means the resident infectious risk index for respiratory diseases, and *H* refers to the risk index of disasters caused by respiratory infectious diseases among urban residents. *E* represents the exposure risk index of residents to the disease, and *V* expresses the vulnerability of residents. Therefore, the specific evaluation value of the risk is calculated by Equation (2) based on the vulnerability, exposure, and upper respiratory tract infectious risk:

(2) $$R_{i}=E_{i}W_{e}\times V_{i}W_{v}\times H_{i}W_{h}$$

In Equation (2), the *E*, *V*, and *H* are calculated by Equations (3)–(5):

(3) $$E_{i}=\overset{n}{\underset{i=1}{\sum }}K_{ei}W_{ei}$$

(4) $$V_{i}=\overset{n}{\underset{i=1}{\sum }}K_{vi}W_{vi}$$

(5) $$H_{i}=\overset{n}{\underset{i=1}{\sum }}K_{hi}W_{hi}$$

$R_{i}$ is the residents’ comprehensive infectious risk against respiratory diseases in the i community. The larger the value, the greater the community residents’ infectious risk is. In addition, $E_{i}$, $V_{i}$, and $H_{i}$, respectively, represent the residents’ exposure, vulnerability, and upper respiratory tract infectious risk in the i community; $K_{ei}$, $K_{vi}$, and $K_{hi}$ demonstrate the quantities of the three indicators, respectively; $W_{ei}$, $W_{vi}$, and $W_{hi}$ express the weights of the three indicators, respectively.

Further, original data with different degrees and quantification will be standardized to eliminate the quantitative relationship between different variables and make comparison easier. To this end, Equations (6) and (7) are used to screen all data indicators according to the positive and negative indicators.

(6) $$x_{i}=\frac{x_{i}}{\max\left(x_{i^{`}}\right)}$$

(7) $$x_{i}=\frac{\min(x_{i^{`}})}{x_{i^{`}}}$$

Equation (6) is a positive index operation, and Equation (7) is a negative index operation, where $x_{i}$ and $x_{i^{`}}$ represent the standard value and the original value of the ith index. max($x_{i^{`}}$) and min($x_{i^{`}}$) denote the maximum and minimum value of the ith index, respectively.

Subsequently, PSO will use the original high-level data characteristics to calculate the index score. The main steps are: 1. the population with different conditions is divided; 2. the most suitable position is found according to the conditions; 3. the particle swarm is initialized; 4. the fitness of the current particle position is calculated; 5. the current position of the particle is set as Pbest; 6. the position Gbest with the highest fitness is taken; 7. a questionnaire survey is issued to the community with the highest fitness, and 8. the best risk assessment is obtained by summarizing. The quickest way for a bird swarm to search for food randomly is to infer the individual closest to the food source if there is only one food source in this specific area. In this “bird flock”, individuals communicate to acquire new knowledge and change their behaviors according to the environmental situation [16]. The evolution of the entire bird population includes the birth of new organisms, the process of group classification, and the process of searching for food.

There are a total of *n* particles in the space of dimension *d*, and each particle has a potential solution obtained by the objective function. The fitness is the function value of the particle position, which is used to judge the quality of the particle position. Equations (8) and (9) display the particle’s position and update speed:

(8) $$v_{id}\left(t+1\right)=W*v_{id}\left(t\right)+c_{1}r_{1}\left(P_{id}-X_{id}\left(t\right)\right)+c_{2}r_{2}\left(P_{gt}-X_{id}\left(t\right)\right)$$

(9) $$X_{id}\left(t+1\right)=X_{id}\left(t\right)+v_{id}\left(t+1\right)$$

$v_{id}\left(t+1\right)$ is the velocity of particle *i* after *t* + 1 iteration; W is the weight of particle velocity; $v_{id}\left(t\right)$ is the velocity of *i* in dimension *d* at iteration *t*; $X_{id}\left(t\right)$ is the position of *i* in dimension *d* of the *t*th iteration; $c_{1}$ and $c_{2}$ indicate learning factors and are positive real numbers, and $r_{1}$ and $r_{2}$ are random distribution functions on the interval [0, 1].

### 2.3. Community Space Structure Planning for Healthy Cities

**Figure 3:**
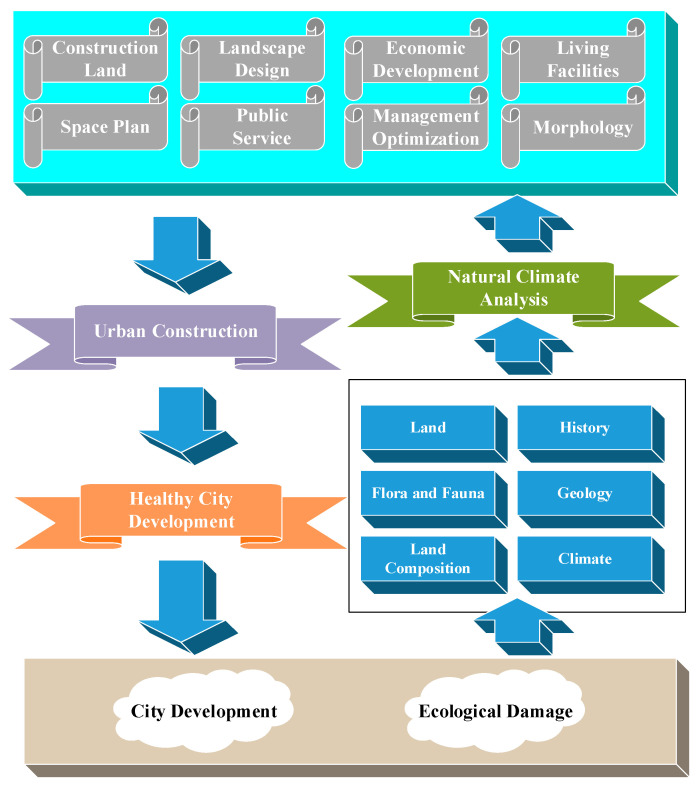
Healthy city’s community spatial structure planning.

A healthy city focuses on people’s health in all aspects, from urban planning, construction, and management to ensure the healthy life and work of the general public. The health city is an organic integration of healthy people, environment, and society necessary for the development of human society [17,18]. A healthy city’s space planning is to regenerate urban resources and redistribute urban materials. Rational construction of land planning can help adjust the overall urban spatial design to promote sustainable city growth. It is a predetermined procedure for controlling land use and planning for fair welfare. The purpose of urban planning is to realize residents’ high-quality life through urban construction and other strategies and initiatives. From another perspective, urban planning is to determine the size, nature, and development direction of a city. Planning the urban physical space achieves the economic and social development goals over a certain period of time and promotes the quality of life of the urban population [19,20]. Figure 3 shows the healthy city’ community spatial planning structure.

**Figure 4:**
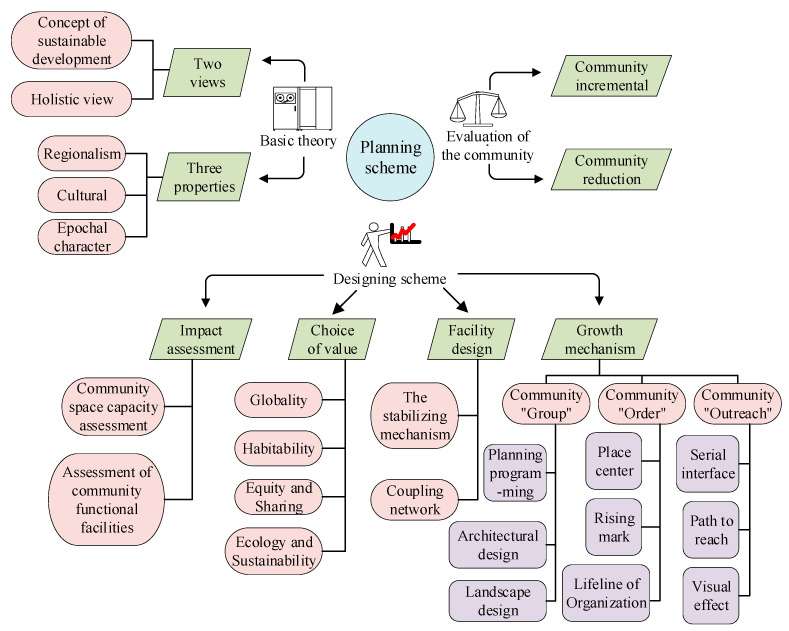
The healthy city community space’ specific planning process and schemes.

The specific planning scheme of the healthy city community space is presented in Figure 4:

The proposed community space planning scheme plans not only material resources and visualizes functions through smart technologies but also covers various cultural factors. The proposed community spatial structures center around resents’ health and offer a novel community space planning.

The following factors influence the planning and management of the community spatial structure:

1. Rational organization of urban road traffic. In particular, the division of arterial roads is often the main factor determining the scale of residential land.
2. The radius of public facilities for residents living services. Reasonable facility scale (i.e., the economy of operation and management) and service radius (i.e., the walking distance of residents using public buildings) affect the convenience and safety of residents’ lives.
3. A unified urban administrative management system. As the basic cell of society, the family is bound to be linked with the administrative organization of society.
4. Terrain. The organizational form of residential areas is affected and restricted by topography to a certain extent. It is generally difficult to compactly arrange residential land on undulating terrain [21].

**Figure 5:**
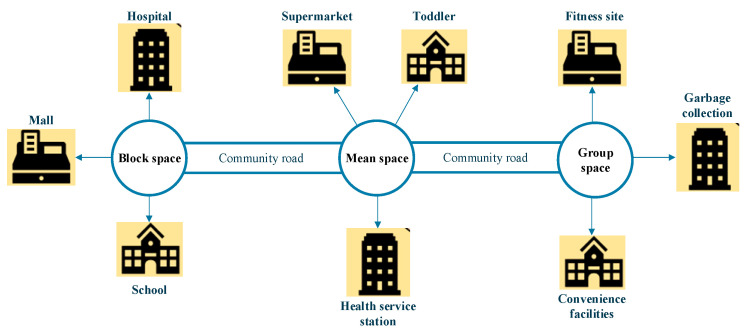
Community three-level prevention and control system.

According to the concept of “three areas and two channels”, healthy urban planning divides the community space into three levels: block, residential area, and group, with public roads running through them. The layout of the spatial form features an overall openness and specific enclosedness. Under such a layout, when respiratory diseases spread, the community space can be rapidly transformed into a three-level prevention and control system [22], as shown in Figure 5.

In Figure 5, the block space is mainly an open mode, providing the public with municipal, commercial, transportation facilities, and other services; it serves within the block and also serves its neighbors, realizing the fully open and shared 15 min living circle. The residential space is mainly semi-open to create good lighting, ventilation, and a green environment; it sets up adequate outdoor fitness places for vulnerable groups and susceptible people to carry out basic fitness activities downstairs. The group space is mainly shared in private mode, and the convenience facilities, public elevators, corridors, garbage collection facilities, and other public service supporting facilities within are group-specific services, not shared externally [23].

Different from the traditional layout of the residential area, the community’s three-level spatial layout can further divide the closed level of the residential area according to the road grade. “Sina” is a new community pilot project. “Sina”, an overall openness and specific enclosedness pattern, increases the space for neighborhood communication and can be relatively enclosed to form isolated units under abnormal conditions. Meanwhile, the medical concept of “three areas and two channels” is combined in the plane layout. It follows the design principles of the healthy city in detail design and relies on the intelligent system in management. The layout equips the community with the resilience to handle sudden crises and realizes the original intention of “managing and controlling” an open community [24].

### 2.4. Reliability and Validity Analysis

Reliability means the consistency or homogeneity of the measurement results. According to the literature, reliability can be divided into internal reliability and external reliability. Internal reliability refers to whether a group of questions measures the same concept, that is, how consistent is the group of questions. Common analysis methods include Cronbach’s and split-half reliability. External reliability refers to the degree of consistency of repeated measurements on the same research object for the same questionnaire at different times. The commonly used analysis method is retesting by SPSS. Validity is to test the validity of the questionnaire; generally speaking, it is to determine whether the designed items are reasonable and whether they can effectively reflect the researcher’s goals. A questionnaire survey usually contains single-choice, multiple-choice, fill-in-the-blank questions, and other forms of questions. Validity analysis is only for scale data, and non-scale items such as multiple-choice, single-choice gender, and other items cannot be used for validity analysis [25].

### 2.5. Questionnaire Design of Investigating Healthy City Resident’s Health Status and Public Services

As a developing country, China still struggles to transform all community health services into public institutions in the short term. Since 2008, Hefei City has greatly improved the environment and facilities of community health service institutions through innovation and increasing investment in community health services. In November 2011, 127 community health service institutions were established in the urban area of Hefei, and the coverage rate of community health service for urban residents was over 95% [26]. This experiment conducted an online questionnaire survey on the residents of the smart community in Hefei, and the distribution objects were divided into the elderly, the middle-aged, the adolescent, the sick (including various physical diseases), and the healthy group. The questionnaire survey investigated residents’ health, mainly from respiratory diseases and community health services. Respiratory diseases are based on the residents’ basic knowledge of pneumonia, community epidemic prevention work, self-vaccination, and physical conditions. Community service involves the community environment, community staff, work content, and physical and mental health status. Table 2 exhibits the content of the questionnaire:

The validity of the questionnaire needs to be judged by a specific validity test. The validity of the questionnaire is tested by statistical software. The relevance of the survey topic, the correctness of the question, and the appropriateness of the survey time are tested based on the obtained initial data. Through the evaluation of statistical methods and relevant experts, the obtained results of the validity test are expressed in Table 3.

According to Table 3, the overall effect and performance of the questionnaire design were good, and the validity was up to standard. Follow-up data collection can be continued. The questionnaire was distributed twice: before the implementation of the proposed HCC-oriented community spatial planning structure and after the implementation. Overall, 200 copies of questionnaires were distributed to residents in the community in a healthy city in a certain place, and 186 valid questionnaires were collected, with an effective rate of 93%.

## 3. Results

### 3.1. Analysis of the Daily Living Ability of Patients with Respiratory Diseases

This section uses a questionnaire survey to analyze before and after the proposed HCC-oriented community spatial planning structure. Community services are investigated from the aspects of a community environment, community staff, community service work content, and physical and mental health status. A scoring system is adopted for statistical analysis, and each item is scored from 0 to 30 points. The results will reflect the patients’ physical mobility and their ability to perform activities of daily living. The questionnaire data needs to be sorted and analyzed before processing. In this experiment, the questionnaire is divided into two aspects for investigation. The first is a survey on the disease status of community patients with respiratory diseases (mainly COVID-19). In the experiment, the effectiveness of the proposed HCC-oriented community spatial planning structure was identified by comparing the scores of patients’ ability to act by themselves and scores of their daily life impact before and after the implementation of the proposed community structure. The relevant data in the questionnaire is drawn as a statistical graph for analysis of the results. The data results are illustrated in Table 4.

**Figure 6:**
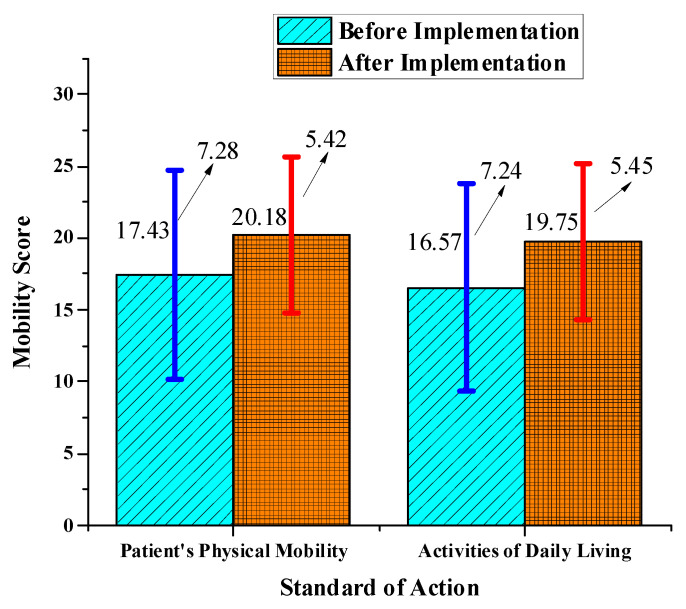
Comparison of daily activity scores of community patients with respiratory diseases before and after the implementation of the proposed community structure.

The total score of the statistical questionnaire is 2312 before the implementation of the proposed community structure and 2715 after the implementation. Apparently, the service quality of residents after the implementation has improved. The analysis of the two modules: epidemic prevention and community health services, shows that both before and after implementation, epidemic prevention work has higher scores than daily work. Thus, the community is relatively secure. However, it also manifests that while the service workers are increasing their efforts to overcome the epidemic, they are negligent in daily health services. Therefore, these public servants are expected to pay more attention to managing community health, coordinate their work, and take corresponding incentive measures. The data results are expressed in Figure 6.

Figure 6 manifests that before the implementation of the proposed community structure, the average score of physical mobility of patients with respiratory diseases in the community was 17.43, and the score of positive and negative errors was 7.28. After the implementation, the score of mobility increased to 20.18, and the score of positive and negative errors has also been reduced to 5.42. Thus, the HCC-oriented community spatial planning has improved the physical self-control ability of patients with respiratory diseases and helped patients reduce the pain of the disease. As for the daily activities of patients, the average score before the implementation of the healthy city was 16.57, and the error in the positive and negative directions was 7.24. After the implementation of the proposed community structure, the score of activities increased to an average of 19.75, and the score of error in the positive and negative directions was reduced to 5.45. The results showed that the overall daily activities of patients were improved. It can be observed that the planning and optimized management of a healthy city can greatly reduce chronic patients’ pains, improve their daily activities, and encourage their normal lives. Overall, the finding reflects the importance of HCC.

### 3.2. Community Health Services Coverage among Healthy Cities’ Residents

**Figure 7:**
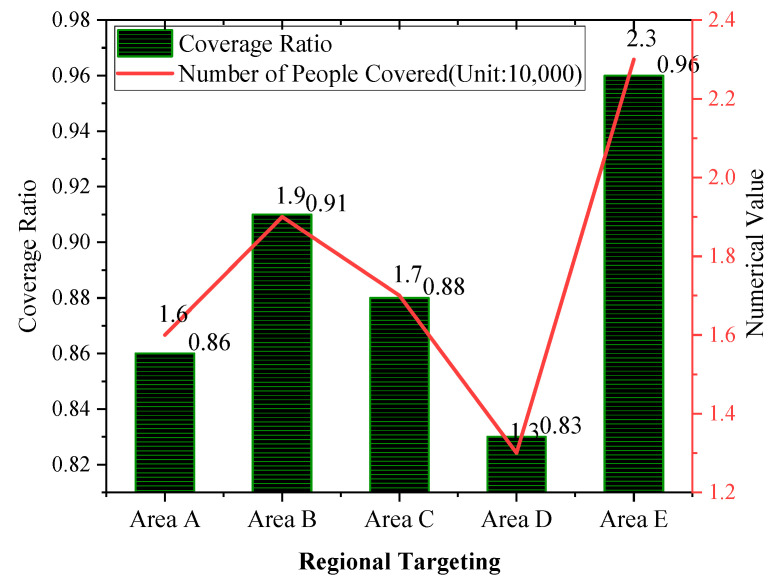
Healthy city residents’ community healthy service coverage.

As previously completed, the chronic patients are evaluated on their self-control ability and the scores of daily activities. It is found that the proposed HCC-oriented community spatial planning can indeed improve chronicle patients’ self-care ability and is thus effective. Therefore, this section will analyze the second part of the questionnaire, that is, the healthy city’s resident’s community health service coverage. The number of people who knew the community health service planning of healthy cities in the questionnaire survey is counted, and the proportion in the total number of respondents is calculated. Similarly, the results of the questionnaire will be drawn into a statistical graph, which is indicated in Figure 7.

According to Figure 7, among regions A to E, the coverage of health services in community E is the highest, reaching 96%, and about 23,000 people can enjoy health security services, followed by region B, with a coverage rate of 91%, covering 19,000; followed by region C, with a coverage rate of 88%, including 17,000; followed by area A, with a coverage rate of 86%, covering about 16,000 people, and finally community D, with a coverage rate of only 83% and involving about 13,000 people. Theoretically, health security services in a healthy city should be a fundamental resident right. Nevertheless, so far, no community has reached 100% coverage. Therefore, more work is needed in HCC to reach 100% healthy service coverage, which is the basic requirement of a healthy city.

## 4. Discussion

The questionnaire survey on residents’ health status and community health service comprehensively reveals the research results. First, the implementation of the proposed HCC-oriented community spatial planning can help patients alleviate pain, advance their self-control ability, and improve their daily activities. The proposed community structure has brought great benefits to residents’ lives during the epidemic and sets an example for all communities to follow and practice. Second, the coverage of community health security is relatively high, reaching 88%. It indicates the vast majority of community health services are guaranteed. Due to varying factors, the investigation and cooperation of relevant departments and every citizen are required in HCC. HCC calls for more, and the following suggestions might help to accelerate HCC:

1. The provision of community health and medical personnel should be accelerated. Community health medical personnel are the foundation of the development of community health services. Without excellent personnel, there will be no quality service. General practice and community nursing programs can be established in medical colleges and universities to specifically train professionals suitable for community health [27].
2. A sound community health electronic information platform needs to be established. The informatization of community health service has played an important role in promoting the development and mode transformation of community health.
3. The income of employees should be increased, and a scientific performance appraisal system should be implemented. While implementing two lines of revenue and expenditure, a positive and effective performance appraisal system should be adopted. Otherwise, the efficiency of community health services will be inevitably affected [28].

In this way, healthy cities should be vigorously developed, and optimized management and health services in the community environment should be strengthened to prevent and control the epidemic better and safeguard residents’ life and security.

## 5. Conclusions

The raging COVID-19 affects both China and countries worldwide, and it is said to coexist with humans for a long time. In this context, this work discusses how to develop a healthy city and optimize its management. Firstly, the theoretical sources of the healthy city are summarized together with its cognitive stages in human history and its main functions and completion standards. Secondly, a PSO is chosen to calculate the healthy city residents’ health index. PSO can directly solve the best solution by combining the original high-level data characteristics. Finally, an HCC-oriented community spatial planning structure is proposed and tested through a questionnaire survey. The questionnaire survey tries to understand the patients’ living ability and the community health security coverage under the influence of the pandemic. The following two results are obtained. (1) After the implementation of the proposed community structure, patients’ daily activities have been greatly improved. (2) At present, the community residents’ health security coverage has reached 88%. Then, corresponding recommendations are proposed based on the survey results. Last but not least, there are still some shortcomings in this experiment. The questionnaire questions are not comprehensive enough and need improvement. Therefore, in the follow-up research, the interview scope of the questionnaire will be expanded, and the generality of the experiment will be improved.

## Figures and Tables

**Table 1:** Difficulties in creating healthy cities.

City Disease	Influence Situation
Polluted environment	Residents have low health conditions and poor living conditions.
Mass infection	
Traffic congestion	Residents’ simple but basic needs are not always met, such as traveling and some living materials.
Stressful life	
Frequent disasters	Job opportunities are rare, and defaults on wage payments occur occasionally.
Prominent social problems	

**Table 2:** The questionnaire.

**Basic Information**								
Age		Do you have a chronic disease?						
Gender		Have you been infected with COVID-19?						
No.	Content of the questionnaire			Options: A. Strongly agree B. Relatively agree C. Neutral D. Disagree E. Extremely disagree				
**Respiratory diseases (mainly COVID-19)**								
1	COVID-19 is extremely contagious.			A	B	C	D	E
2	Maintaining personal and environmental hygiene is an effective measure to prevent COVID-19.			A	B	C	D	E
3	In a crowded environment, I always wear a mask.			A	B	C	D	E
4	I am actively vaccinated against COVID-19.			A	B	C	D	E
5	You are satisfied with your community’s COVID-19 prevention efforts.			A	B	C	D	E
6	Have you recently contracted other respiratory diseases?			A	B	C	D	E
7	Community workers will often visit your home to check on your health.			A	B	C	D	E
**Status of community safety and health services**								
1	I think my living environment is clean and up to standard regarding hygiene.			A	B	C	D	E
2	I can achieve my goals in my living environment in a healthy state.			A	B	C	D	E
3	My life and activities will not be threatened by environmental sanitation.			A	B	C	D	E
4	Community staff will conduct regular health and hygiene training.			A	B	C	D	E
5	You are satisfied with the health service attitude of the community workers.			A	B	C	D	E
6	Community workers can achieve health protection purposes in a variety of ways.			A	B	C	D	E
7	You are satisfied with the work content of health care in the community.			A	B	C	D	E
8	You think the spread of healthy communities is very important.			A	B	C	D	E
9	The appearance of community workers is decent.			A	B	C	D	E
10	The community environment is quiet, clean, and human-focused.			A	B	C	D	E
11	Community service workers are very concerned about the residents, and they can help answer any concerns in a timely manner.			A	B	C	D	E
12	Community health service centers are generally trustworthy.			A	B	C	D	E
Other supplements:								

**Table 3:** Results of the validity test of the questionnaire.

	Methods	Statistics	Expert Assess
Performance			
Initial data		Good	Good
Relevance		Good	Good
The correctness of the question		Good	Better
The appropriateness of the survey time		Good	Good

**Table 4:** Comparison of scores of daily activities of patients with community respiratory diseases before and after the implementation of the proposed HCC-oriented community spatial planning structure.

	Judgment Standard	Patient’s Physical Mobility	Daily Activities
Time of Evaluation			
Before the implementation of the proposed community structure		17.43 ± 7.28	16.57 ± 7.24
After the implementation of the proposed community structure		20.18 ± 5.42	19.75 ± 5.45

## Data Availability

Not applicable.
